# The effects of a cyclooxygenase-2 (COX-2) expression and inhibition on human uveal melanoma cell proliferation and macrophage nitric oxide production

**DOI:** 10.1186/1477-3163-6-17

**Published:** 2007-11-27

**Authors:** Jean-Claude Marshall, Amanda L Caissie, Stephanie R Cruess, Jonathan Cools-Lartigue, Miguel N Burnier

**Affiliations:** 1 The Henry C. Witelson Ocular Pathology Laboratory and Registry, McGill University, 3775 University Street, Lyman Duff Building, Room 216, Montreal, Quebec H3A 2B4, Canada

## Abstract

**Background:**

Cyclooxygenase-2 (COX-2) expression has previously been identified in uveal melanoma although the biological role of COX-2 in this intraocular malignancy has not been elucidated. This study aimed to investigate the effect of a COX-2 inhibitor on the proliferation rate of human uveal melanoma cells, as well as its effect on the cytotoxic response of macrophages.

**Methods:**

Human uveal melanoma cell lines were transfected to constitutively express COX-2 and the proliferative rate of these cells using two different methods, with and without the addition of Amfenac, was measured. Nitric oxide production by macrophages was measured after exposure to melanoma-conditioned medium from both groups of cells as well as with and without Amfenac, the active metabolite of Nepafenac.

**Results:**

Cells transfected to express COX-2 had a higher proliferation rate than those that did not. The addition of Amfenac significantly decreased the proliferation rate of all cell lines. Nitric oxide production by macrophages was inhibited by the addition of melanoma conditioned medium, the addition of Amfenac partially overcame this inhibition.

**Conclusion:**

Amfenac affected both COX-2 transfected and non-transfected uveal melanoma cells in terms of their proliferation rates as well as their suppressive effects on macrophage cytotoxic activity.

## Introduction

Uveal melanoma is the most common primary intraocular tumor in adults. The ability of ophthalmologists to diagnose the primary tumor has increased from an average accuracy of 87.5 percent in 1980 [1] to approximately 99.5 percent in 1990 [2]. This increase in diagnostic accuracy reflects better training, and the introduction of new clinical tools such as A and B scan ultrasound. As diagnosis has improved so too has local treatment, with the development of radiotherapy a much more conservative option than the previous standard treatment of enucleation. There is, however no difference between mortality rates of patients treated with either of these local therapies [3]. A patient who now presents with this disease continues to have the same 10-year mortality rate of approximately 40% as those who were diagnosed three decades ago [4]. It is therefore apparent that a further understanding of the cellular mechanisms behind this disease and its metastatic processes are required in order to identify novel prognostic factors and targets for systemic therapy that will affect patient prognosis.

Several prognostic factors of uveal melanoma, such as cell type, have been utilized for decades. More recently identified prognostic factors include tumor associated macrophages (TAM) which have been shown to be a predictor of poor prognosis in uveal melanoma [5]. The activity of these TAM in the tumor and the possible immunosuppression of the macrophages by tumour-secreted factors has previously been studied in cutaneous melanoma [6]. The effect of uveal melanoma-secreted factors on the cytotoxic activity of macrophages has not yet been investigated. The demonstration that cyclooxygenase-2 (COX-2) inhibition can reverse melanoma induced suppression of macrophage cytotoxic activity in cutaneous melanoma [6], is of interest as COX-2 expression has recently been identified in uveal melanoma [7].

COX-2 is not only a prognostic factor but also a potential therapeutic target in uveal melanoma. There are three isoforms of the COX enzyme. COX-1 is expressed constitutively in normal tissues [8]. COX-2 is an inducible enzyme expressed in response to a variety of inflammatory and mitogenic stimuli [9]. COX-2 expression has been reported in a wide variety of malignant tumors [10-12], including uveal melanoma [7], where it was correlated with predictors of poor prognosis. The expression of COX-2 has been linked to various processes including tumor proliferation [13], immunosuppression [14] and metastasis [15,16]. Specific COX-2 inhibitors are currently in use for patients diagnosed with familial adenomatous polyposis, a genetic disorder that predisposes patients to colonic adenocarcinomas [17]. The effectiveness of these selective inhibitors has been investigated in a variety of tumors and shows promise for use as an adjuvant therapy in the treatment of many tumor types [18].

The aim of this study was to investigate the possible role of COX-2 expression and inhibition by a COX-2 inhibitor (Nepafenac) on the proliferation rates of human uveal melanoma cell lines. In addition, we wished to investigate the effect of soluble factors secreted by human uveal melanoma cell lines on the cytotoxic activity of macrophages. Amfenac, a COX-2 inhibitor formulated for topical administration to the eye [19,20], was investigated in terms of its effects on macrophage cytotoxicity in response to soluble factors secreted by uveal melanoma cell lines.

## Methods

### Cell Culture

Four previously characterized human uveal melanoma cell lines (92.1, SP6.5, MKT-BR, OCM-1) and one human transformed uveal melanocyte cell line (UW-1) were incubated at 37°C in a humidified 5% CO_2_-enriched atmosphere [21]. The cells were cultured in RPMI-1640 medium (Invitrogen, Burlington, Ontario, Canada), supplemented with 5% heat inactivated fetal bovine serum (FBS), 1% fungizone, and 1% penicillin-streptomycin purchased from Invitrogen (Burlington, Ontario, Canada). Cells were cultured as a monolayer in 25 cm^2 ^flasks (Fisher, Whitby, Ontario, Canada) and observed twice weekly, at every media change, for normal growth by phase contrast microscopy. The cultures were grown to confluence and passaged by treatment with 0.05% trypsin in EDTA (Fisher) at 37°C and washed in 7 ml RPMI-1640 media before being centrifuged at 120 g for 10 minutes to form a pellet. Cells were then suspended in 1 ml of medium and counted using the Trypan Blue dye exclusion test for use in all subsequent assays.

The uveal melanoma cell lines 92.1, SP6.5, and MKT-BR were established by Dr. Jager (University Hospital Leiden, The Netherlands), Dr. Pelletier (Laval University, Quebec, Canada) and Dr. Belkhou (CJF INSERM, France), respectively. Dr. Albert (University of Wisconsin-Madison, USA) established the OCM-1 and UW-1 cell lines [22,23].

### Transfection with COX-2

The four human uveal melanoma cell lines and one transformed melanocyte cell line were transfected to constitutively express COX-2 using lipofectAMINE^® ^as per the manufacturer's recommendations (Invitrogen). Briefly, 1 × 10^5 ^cells were seeded in a 10 cm petri dish. Cells were then allowed to incubate overnight. The pcDNA3 plasmid (Invitrogen) encodes for resistance to Geneticin. This plasmid, with COX-2 cDNA inserted at the XBA I cleavage site, was used at a concentration of 2 ug per 10 ul of LipofectAMINE in 100 ul of serum-free OptiMem medium (Invitrogen). The cells were incubated for four hours with this solution. After removal of the solution, cells were grown in 5% FBS supplemented RPMI-1640 medium with 400 ug/ml of Geneticin (G418, Gibco). Cells were also transfected with the empty pcDNA3 plasmid and compared to the original non-transfected cell lines as controls.

### Western blot and Immunohistochemistry

COX-2 expression was verified by Western blot and immunohistochemistry performed on cytospins of the five cell lines.

Western blot analysis was done as previously described [24]. Briefly, protein samples from the cell lines were prepared using 100 ul of 2× electrophoresis sample buffer (250 mM TRIS pH 6.8, 4% SDS, 10% glycerol, 0.006% bromophenol blue, 2% beta mercaptoethanol) per million cells, which was then boiled for 5 minutes. The same number of cells per sample was used. Proteins were separated on 12% SDS-Page gel and transferred to a polyvinylidene difluoride membrane overnight (Amershame bioscience, Piscataway, NJ, USA). The membrane was blotted for specific antibody according to Proto Blot for Western blot alkaline phosphatase system (Promega Corporation, Ontario, Canada). The primary antibody, monoclonal mouse anti-human COX-2 (Zimed Laboratories, San Francisco, CA, USA; clone COX 229), was used at a concentration of 3 ug/ml. The secondary, a goat anti-mouse alkaline phosphatase-conjugated antibody (Sigma-Aldrich, Ontario, Canada) was used to visualize the proteins on the membrane. Cell lysate from macrophages stimulated with interferon gamma and LPS (Transduction Laboratories, BD Biosciences, San Diego, CA, USA) was used as a positive control. A broad range molecular weight marker (BioRad) was used. Equal protein loading of cell extracts in SDS-Page was determined by Bio-Rad protein assay solution.

The cytospin slides were removed from -20°C and left at room temperature overnight before being fixed with 2% paraformaldehyde for 30 min. The paraformaldehyde-fixed samples were then tested for COX-2 expression by immunocytochemical analysis using a monoclonal mouse anti-COX-2 antibody (Zimed Laboratories, San Francisco, CA, USA; clone COX 229) at a dilution of 1:50. Immunohistochemistry was performed using the Ventana BenchMark fully automated staining machine with a sample of cutaneous melanoma as a positive control.

### Proliferation assay

The Sulforhodamine-B based assay (TOX-6, Sigma-Aldrich, St. Louis, Missouri, USA) was performed according to the National Cancer Institute protocol [25]. Each of the transfected and non-transfected cell lines was seeded into wells at a concentration of 2.5 × 10^3 ^cells per well, with a minimum of six wells per cell line. A row of 8 wells containing only RPMI-1640 medium was used as a control. Cells were allowed to adhere overnight. The active metabolite of Nepafenac, amfenac, was added to each well at its IC50 of 150 nM [26]. Cells were then allowed to incubate for 48 hours following the addition of Amfenac. Following this 48-hour period, cells were fixed to the bottom of the wells using a solution of 50% Trichloroacetic acid (TCA) for 1 hour at 4°C. Plates were then rinsed with distilled water, to remove TCA and medium and air dried. The Sulforhodamine-B dye solution was then added to each well and allowed to stain for 25 minutes. The Sulforhodamine-B solution was subsequently removed by washing with a 10% acetic acid solution and once again allowed to air dry. The dye that had become incorporated into the fixed cells at the bottom of the wells was solubilized in a 10 mM solution of Tris. The absorbance of the solute was measured using a microplate reader at a wavelength of 510 nm. Cells transfected with only the pcDNA3 plasmid were used as controls and did not significantly differ from the original non-transfected cell lines.

For flow cytometry cell cycle analysis, cells were passaged and counted as described previously [27]. Cell pellets were resuspended in 1 ml of RPMI 1640 medium at a concentration of 1 × 10^6 ^cells/ml. The cells were labelled with propidium iodide and a measurement of the cellular DNA was then performed using an Epics XL flow cytometer (Beckman Coulter, Miami, Florida). The percent of cells in the S phase fraction (SPF) were then recorded for each of the three replicates and averaged together.

### Macrophage NO Production Assay

The four human uveal melanoma cell lines (92.1, MKT-BR, OCM-1, SP6.5) and the transformed melanocyte cell line (UW-1) with and without COX-2 transfection, as well as one monocyte cell line (28SC), were seeded in 6 well plates at a concentration of 1 × 10^6 ^cells/ml in 5% FBS supplemented RPMI-1640 medium. Eighteen hours after seeding the melanoma cells, medium was removed from each well and centrifuged at 120 g for 10 minutes to remove cells. The supernatant was then used as melanoma conditioned medium (MCM). 28SC was then exposed to MCM from the transfected and non-transfected cell lines with and without amfenac at the recommended IC_50 _of 150 nM. 28SC incubated in fresh medium was used as control. Twenty-four hours after media transfer, 28SC were stimulated with 100 U/ml of Interferon gamma (IFNγ) and 100 ng/mL of lipopolysacharide (LPS). The experiment was done in triplicate. After 24 hours of stimulation supernatants were removed and assayed for nitrite levels using the Greiss reaction. One hundred μl of medium was reacted with an equal mixture of 1% sulphanilamide and 0.1% naphthylethylenediamine dihydrochloride in 2.5% phosphoric acid. The mixture was incubated at room temperature for 10 minutes and then the absorbance was read at 550 nm using a BioTek EL 800 microplate reader (Fisher). Readings were compared to a standard curve to calculate nitrite levels.

### Statistical Analysis

The Student's t-test was used to compare proliferation results between transfected and non-transfected cell lines, as well as the same cell lines exposed to amfenac. It was also used to compare results of 28SC incubated with conditioned medium from COX-2 transfected versus non-COX-2 transfected melanoma cell lines as well as 28SC incubated with conditioned medium from these cell lines with and without amfenac. Flow cytometry results were compared using a one-way ANOVA test with LSD post hoc analysis

## Results

### COX-2 expression

**Figure 1:**
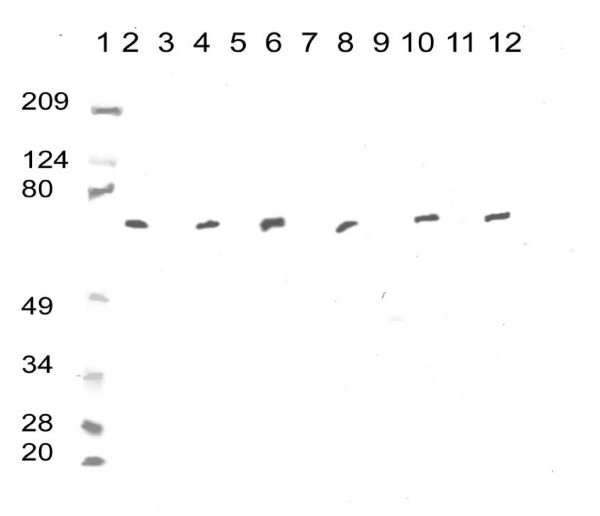
Western blot showing COX-2 expression. Lane 1: molecular weight marker, weights are given in kilo daltons, lane 2 the positive control for COX-2 at 70 kDa, lane 3 the 92.1 cell line, lane 4 the 92.1 transfected cell line, lane 5 the MKT-BR cell line, lane 6 the transfected MKT-BR cell line, lane 7 the OCM-1 cell line, lane 8 the transfected OCM-1 cell line, lane 9 the SP6.5 cell line, lane 10 the transfected SP6.5 cell line, lane 11 the UW-1 cell line, and lane 12 the transfected UW-1 cell line.

Neither the five human uveal melanoma cell lines nor the macrophage cell line constitutively expressed detectable levels of COX-2 as measured by western blot analysis (Figure 1) or immunohistochemsitry. All uveal melanoma cell lines were positive for COX-2 expression following transfection, with no apparent difference in expression between the five transfected cell lines.

### Proliferation Assays

**Figure 2:**
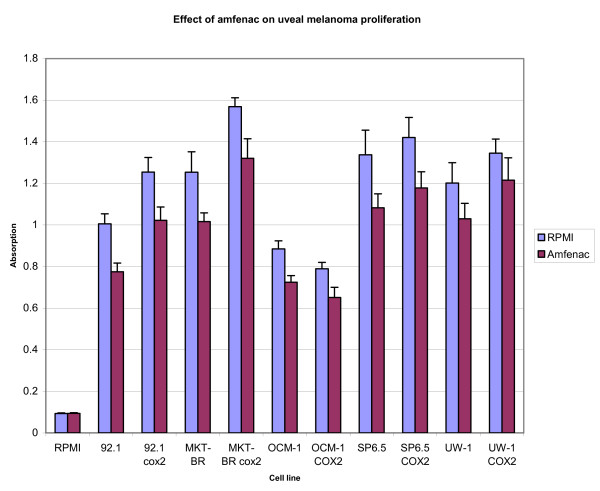
Graph of proliferation rates of four human uveal melanoma cell lines (92.1, MKT-BR, OCM-1, SP6.5) and the UW-1 transformed melanocytic cell line, with and without addition of amfenac. RPMI alone was used as a control.

With the Sulforhodamine-B assay, an increase (p < 0.05) in proliferation was seen in four of the cell lines expressing COX-2 (92.1, MKT-BR, SP6.5, UW-1), while a decrease in proliferation was seen in one cell line (OCM-1) as compared to their non-transfected counterparts (p < 0.05). The addition of amfenac inhibited the proliferation of all cell lines (p < 0.05), with an inhibition that ranged between 20–22% for all cell lines including OCM-1. Figure 2 shows the difference in proliferation rate between the COX-2 expressing and non-transfected cell lines as well as the decrease in proliferation for all cell lines treated with amfenac. The empty pcDNA3 plasmid transfected cells did not show any change in proliferation as compared to the original cell lines.

The percentage of cells in the S phase fraction (SPF%) is shown in Table 1. A statistically significant (p < 0.05) increase in SPF% was seen for each cell line that was transfected to express COX-2. There was a decrease SPF% for each cell line exposed to amfenac, with a larger decrease in SPF% seen in those cells transfected to express COX-2.

**Table 1:** The fraction of cells in S phase as measured by flow cytometry (± standard deviation) for both the original cell line and the COX-2 transfected cell line with and without exposure to amfenac.

**Cell Line**	**Control SPF%**	**SPF% with Amfenac**
**92.1**	14.9 ± 0.8	11.2 ± 0.1
**92.1 COX-2**	30.2 ± 1.2	12.1 ± 0.6
**MKT-BR**	15.7 ± 0.6	13.2 ± 0.4
**MKT-BR COX-2**	29.6 ± 2.1	14.2 ± 0.6
**OCM-1**	18.4 ± 1.6	17.1 ± 1.2
**OCM-1 COX-2**	19.3 ± 0.9	15.8 ± 1.3
**SP6.5**	13.6 ± 0.7	11.3 ± 0.8
**SP6.5 COX-2**	41 ± 3.8	18.9 ± 0.2
**UW-1**	12.5 ± 1.1	10.2 ± 0.9
**UW-1 COX-2**	34.6 ± 2.5	15.8 ± 1.5

### Macrophage NO Production Assay

As compared to control medium, MCM from the four uveal melanoma cell lines significantly reduced macrophage production of NO (p < 0.001). The addition of conditioned medium from UW-1, the transformed melanocytic cell line, yielded no significant decrease in NO production by macrophages. Different levels of macrophage NO production were seen with the MCM from different uveal melanoma cell lines. As a result the different MCMs could be ranked according to their differential effects on macrophage NO production (most macrophage NO production UW-1 > OCM-1 > MKT-BR > 92.1 > SP6.5 least macrophage NO production).

As compared to control medium, conditioned medium from the uveal melanoma cell lines transfected to express COX-2 also significantly decreased macrophage NO production (P < 0.001). This decrease in macrophage NO production was significantly different than that caused by medium from the non-transfected cell lines. The addition of conditioned medium from the COX-2 transfected UW-1 cell line yielded a significant decrease in NO production (P < 0.001) as compared to control medium. The addition of conditioned medium from the COX-2 transfected UW-1 cell line also yielded a significant decrease in NO production (P < 0.0006) as compared to MCM from the non-transfected UW-1 cell line. The different MCMs from COX-2 transfected cell lines could be ranked according to their differential effects on macrophage NO production (most macrophage NO production OCM-1 > UW-1 > MKT-BR > 92.1 > SP6.5 least macrophage NO production).

**Figure 3:**
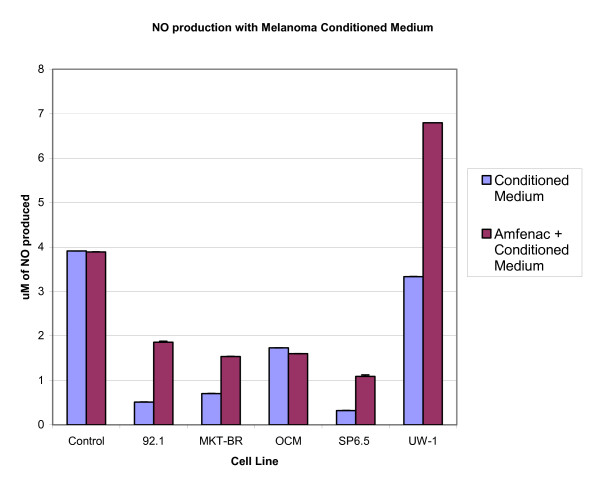
Production of NO by macrophages exposed to melanoma conditioned medium with and without the addition of amfenac.

**Figure 4:**
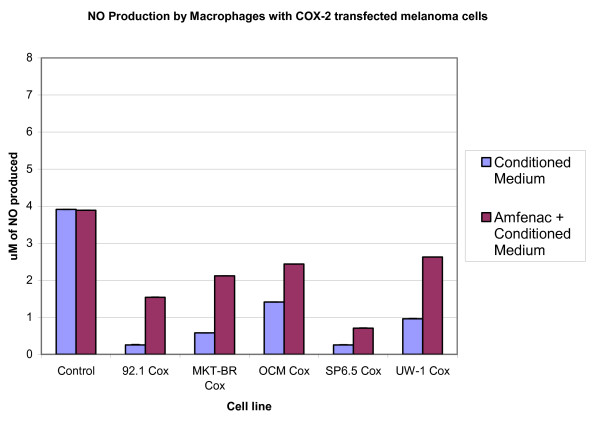
Production of NO by macrophages exposed to conditioned medium from COX-2 transfected cell lines with and without the addition of amfenac.

The addition of amfenac to the MCM of the four uveal melanoma cell lines resulted in increase in macrophage NO production as compared to MCM without amfenac from the same cell lines. This increase was seen with MCM from both transfected and non-transfected uveal melanoma cell lines, with the exception of the non-transfected OCM-1 cell line. The addition of amfenac to the MCM of both transfected and non-transfected UW-1 cells caused an increase in macrophage NO production as compared to MCM without amfenac from the same cell lines. Figures 3 and 4 show the relative macrophage NO production with MCM from both transfected and non-transfected uveal melanoma cells, with and without the addition of Amfenac.

## Discussion

COX-2 expression has been identified in a wide variety of human malignant tumors [10-12], including uveal melanoma [7]. In previous work we have shown 58% of human uveal melanomas to express COX-2 and this expression was linked to histopathological markers of poor prognosis, such as epithelioid cell type, vascular closed loops and tumor infiltrating lymphocytes [7]. The study by Figueiredo et al. gave insight into potential roles for COX-2 in uveal melanoma, which we have proceeded to investigate in the present *in vitro *studies. We also sought to demonstrate the effects of a COX-2 inhibitor on the proliferation and macrophage cytotoxic activity in response to soluble factors secreted by the tumor cells.

COX-2 has been shown to play a role in proliferation *in vitro *studies of prostate [28] and breast tumors [29]. The present study showed that COX-2 transfected human uveal melanoma cell lines had a higher proliferation rate compared to their non-transfected counterparts with the exception of OCM-1. This increase in proliferation rate could be attributed to a wide range of factors produced by the COX-2 enzyme that could influence proliferation rates, such as PGE_2 _[30]. Further investigation into the possible mechanisms for this decrease in proliferation by OCM-1 after expression of COX-2 is currently underway. It is interesting to note that using the Sulforhodamine-B assay a decrease in proliferation was seen for OCM-1 transfected with COX-2, however a small increase in SPF% was seen when analyzed by flow cytometry. Sulforhodamine-B measures the biomass present in each well which may explain the difference with the flow cytometry results.

The present study also showed that the addition of amfenac, the active metabolite of Nepafenac which is a COX-2 inhibitor [19,20], significantly decreased the proliferation rate of all cell lines. What was perhaps even more interesting was the fact that the addition of amfenac not only decreased the proliferation rate of those cells that expressed COX-2, but also the three cell lines that were found to not express COX-2 after testing by immunohistochemistry, and western blott analysis. This result supports the growing body of literature showing a possible COX-2-independent mechanisms of action for certain COX-2 inhibitors such as Celecoxib [9,31,32]. This lack of COX-2 expression in our non-transfected cell lines, despite COX-2 expression in the primary tumors of patients, may reflect that the full tumor microenvironment is necessary for the expression of COX-2 by these cells.

This study is also the first to investigate the effect of uveal melanoma soluble factors on macrophage cytotoxic potential. In uveal melanoma, the presence of tumor associated macrophages (TAM) has previously been shown to be an independent prognostic factor of poor prognosis [5], suggesting that macrophages are not functioning in an anti-tumor role. *In vitro *conditioned medium studies have shown cutaneous melanoma cells to inhibit macrophage tumoricidal activity [6,33] and that COX-2 inhibition was shown to reverse the melanoma induced suppression of macrophage cytotoxic activity [6]. A major mechanism of cytotoxicity by macrophages is the production of nitric oxide, which can be induced by a variety of stimuli such as T-helper cytokines, bacterial wall component LPS, and interferon γ [6]. For our study, we used both LPS and interferon γ as both have previously been shown to stimulate NO production by macrophages [34].

The addition of melanoma conditioned medium to macrophages significantly inhibited the production of NO by four of the five non-transfected human uveal melanoma cell lines. It is interesting to note that the sole cell line that did not significantly inhibit NO production was UW-1. The addition of conditioned medium from the COX-2 transfected cell lines did not give significantly different results for the four uveal melanoma cell lines. In comparison, however, the UW-1 transfected cell line did give a significant decrease in NO production compared to its non-transfected counterpart. This cell line was established from normal melanocytes whereas the other four cell lines were established from the primary uveal melanoma tumors of patients [22,23]. We suggest that the UW-1 cell line behaved differently due to its origins as a transformed melanocytic cell line. This cell line may never have had to escape immune surveillance, and therefore did not induce a decrease in NO production by macrophages. Further investigation into the difference between conditioned medium from UW-1 and conditioned medium from uveal melanoma cell lines is currently underway.

The inhibitory effect of conditioned medium from the five cell lines used on macrophage NO production was ranked for both the transfected and non-transfected cells. In both cases the inhibition was greatest with the SP6.5 and 92.1 cell lines, while OCM-1 and UW-1 gave the least inhibition, with MKT-BR ranking in the middle. This ranking is the same as the proliferative and metastatic ranking of the cell lines that has previously been described [21], indicating that those cell lines with the highest proliferative and metastatic potential also are the ones with the greatest ability to inhibit macrophage NO production.

We have previously demonstrated that a "cross-talk" exists between uveal melanoma tumor cells and macrophages, causing an up-regulation of several soluble factors such as Vascular Endothelial Growth Factor, Interleukin-6, Hepatocyte Growth Factor, and Melanoma Inhibitory Activity [35-37]. These soluble factors have been shown play a role in processes such as tumor proliferation, invasion and cellular motility. The present study is the first to show an inhibitory effect of uveal melanoma soluble factors on the cytotoxic activity of macrophages. Further investigations are warranted to identify the soluble factors responsible for this uveal melanoma-induced suppression of macrophage function.

When the COX-2 inhibitor amfenac was added to the MCM, the suppression of macrophage function was partially overcome in all instances. The addition of amfenac not only affected macrophages exposed to medium from COX-2 transfected cells, but also macrophages exposed to medium from non-transfected cells. This result again supports the hypothesis that amfenac may have a COX-2-independent mechanism of action.

COX-2 inhibitors show promise for use as an adjuvant therapy in many tumor types. However, there has been recent discussion regarding the safety of systemic COX-2 inhibitors, most notably rofecoxib [38]. In our study, we used amfenac, the active metabolite of Nepafenac. This COX-2 inhibitor was formulated for topical administration and may present with a better systemic safety profile than rofecoxib.

In conclusion, COX-2 expressing uveal melanoma cell lines demonstrated an increase in proliferation over their non-COX-2 expressing counterparts. The anti-COX-2 molecule amfenac inhibited the proliferation rate of both COX-2 expressing and non-expressing uveal melanoma cell lines. In addition, amfenac partially overcame the suppression of macrophage function by conditioned medium from both COX-2 transfected and non-transfected uveal melanoma cell lines. Further trials should be undertaken to study the effect of COX-2 inhibitors as potential adjuncts to standard therapy for uveal melanoma and to investigate the potential COX-2 independent function of amfenac.

## Authors' contributions

In vitro work was done by JM, AC, SC. Statistical analysis was done by VS. All authors read and approved the final manuscript prepared by JM.
